# Challenges and Opportunities in State‐of‐the‐Art Proteomics Analysis for Biomarker Development From Plasma Extracellular Vesicles

**DOI:** 10.1002/pmic.70036

**Published:** 2025-09-16

**Authors:** Panshak P. Dakup, Ivo Diaz Ludovico, Youngki You, Chaitra Rao, Javier Flores, Lisa M. Bramer, Marian Rewers, Bobbie‐Jo M. Webb‐Robertson, Thomas O. Metz, Raghavendra G. Mirmira, Emily K. Sims, Ernesto S. Nakayasu

**Affiliations:** ^1^ Biological Sciences Division Pacific Northwest National Laboratory Richland Washington USA; ^2^ Department of Pediatrics Indiana University School of Medicine Indianapolis Indiana USA; ^3^ Barbara Davis Center For Diabetes School of Medicine University of Colorado Anschutz Medical Campus Aurora Colorado USA; ^4^ Department of Medicine and the Kovler Diabetes Center The University of Chicago Chicago Illinois USA

## Abstract

Extracellular vesicles (EVs) are membrane‐bound particles secreted by cells, playing crucial roles in intercellular communication. The composition of EVs can undergo changes in response to stress and disease conditions, making them excellent biomarker candidates. However, extracting protein information from EVs can be challenging due to their low abundance in complex biofluids and copurification with contaminant proteins and particles. Techniques to enrich EVs have their strengths and limitations, without one being able to purify EVs to complete homogeneity. This can lead to compromised recovery rates and increased complexity, making data interpretation difficult. In this viewpoint article, we explore the concept that better characterization of EV composition, followed by quantification of EV proteins in complex samples, might be a more viable route for biomarker development. Mass spectrometers can provide reproducible deep coverage of the EV proteome, despite sample impurities. This paradigm shift presents opportunities to integrate advanced bioinformatics tools to refine the EV proteome landscape, identify novel biomarkers, and streamline validation processes in biomarker development. By focusing on leveraging technology rather than achieving absolute purity, this approach can transform current practices and open opportunities for robust biomarker discovery. Herein, we highlight not only such opportunities but also challenges to implement this concept.

**Summary:**

- Extracellular vesicles (EVs) have enormous potential as biomarkers of diseases, as they can carry signatures of the cells they are derived from and the pathogenesis process.
- Biofluids, such as blood plasma, are highly complex and contain many components with physicochemical properties similar to those of EVs, making it challenging to obtain pure EV fractions.
- Challenges in obtaining pure preparations represent a main hurdle for studying EVs, and their components are potential biomarkers.
- This article explores the concept of studying EV proteins within complex samples, discussing opportunities and needs to move this field forward.

## 1 Introduction

**FIGURE 1:**
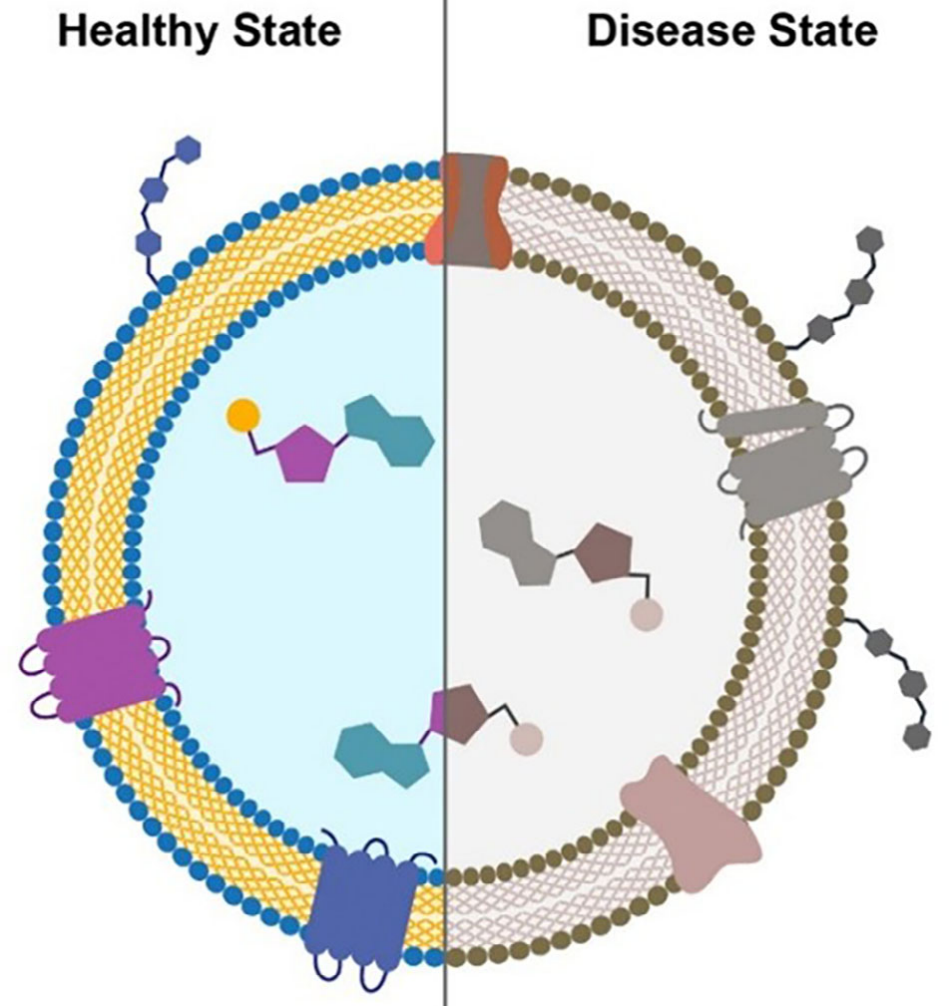
A schematic of an extracellular vesicle (EV) containing proteins that could serve as biomarkers due to changes in composition that reflect the pathological state.

Extracellular vesicles (EVs) are non‐nucleated, lipid bilayer‐delimited particles secreted by cells and composed of lipids, proteins, carbohydrates, metabolites, and nucleic acids [1]. Cells from organisms of all kingdoms secrete EVs [2]. There are two main types of EVs based on their biogenesis: exosomes and ectosomes [1]. Exosomes are EVs of 30–100 nm in diameter that are produced by the invagination of multivesicular bodies, which in turn fuse with the plasma membrane, releasing their contents, including exosomes. Exosomes have some common component markers, such as CD9, CD63, and CD81 [1]. Ectosomes are EVs that form by budding from plasma membranes and exhibit various subtypes. One of the most studied subtypes is microvesicles, also known as microparticles. Microvesicles are typically 100–1000 nm in diameter and may carry markers such as CD40 [1]. EVs have great potential as a source of biomarkers since they carry proteins that are signatures of their cell of origin and disease processes (Figure 1) [3, 4, 5, 6, 7].

In this viewpoint article, we assess EV components are potential biomarkers of diseases. We highlight some of the main challenges in obtaining plasma EV preparations with enough purity and amounts to perform biomarker discovery and validation studies. We discuss an alternative approach of refining the plasma EV composition and quantifying individual components by mass spectrometry (MS), including current limitations and future directions to implement this approach.

## 2 Exploring the Potential for EVs to Serve as Biomarkers

EV components have been explored as biomarkers for a variety of diseases and conditions. Plasma EVs from individuals with Chagas disease identified human leukocyte antigen (HLA) Class I, a well‐characterized EV protein, as a potential biomarker [8]. The study also identified 12 proteins from the causative agent, the protozoan parasite *Trypanosoma cruzi*, as potential biomarkers (Table 1) [8]. In addition, *T. cruzi* epitopes, such as α‐galactose residues and *trans*‐sialidase, have been detected in plasma EVs from infected individuals (Table 1) [9]. This represents proof‐of‐concept that EVs can be excellent sources of biomarkers. In other examples, EV proteins have been identified as biomarkers of pathology for diagnosing and monitoring neurodegenerative diseases. The protein APLP1 was detected in brain‐derived EVs, providing early diagnostic value for brain pathologies (Table 1) [10]. In Parkinson's disease, EV‐associated α‐synuclein has been identified as a diagnostic indicator for individuals at risk for developing the condition [11, 12]. Studies of neuronally derived EVs from serum and plasma have further emphasized α‐synuclein's role in distinguishing high‐risk individuals for Parkinson's, including differentiation between its free protein form and EV‐bound cargo, with potential applications in large‐scale population screening (Table 1) [13, 14]. In distinguishing neurodegenerative‐related conditions, plasma EV levels of TDP‐43 and tau isoforms (3R/4R tau ratios) have been shown to discriminate between frontotemporal dementia and amyotrophic lateral sclerosis (Table 1) [15, 16]. These markers exhibited a strong correlation with neurodegeneration as well as clinical and neuropsychological indicators of disease severity [6, 16].

**TABLE 1:** Summary of EV‐derived biomarkers across diseases.

Study	Disease	EV source	Analytical methods	Key findings
Cortes‐Serra et al. [8]	Chagas disease	Plasma EVs	Proteomics	Identified HLA class I as potential biomarker; 12 *T. cruzi* proteins detected.
Madeira et al. [9]	Chagas disease	Peripheral blood‐derived EVs	ELISA	Detected EV α‐galactose residues and *trans*‐sialidase as biomarkers.
Choi et al. [10]	Neurodegenerative diseases	Plasma EVs	Proteomics, ELISA	APLP1 identified as a novel brain‐derived EV biomarker.
Yan et al. [13]	Parkinson's disease	Plasma EVs	Single EV immunoassay	Detected EV‐associated α‐synuclein as an early‐stage biomarker.
Vacchi et al. [15]	Parkinson's disease	Plasma EVs	Flow cytometry, immunoassay	Identified 11 EV surface antigens as diagnostic biomarkers.
Chatterjee et al. [16]	Frontotemporal dementia and amyotrophic lateral sclerosis	Plasma EVs	Immunoassay	EV TDP‐43 levels and 3R/4R tau ratios discriminate between FTD & ALS.
Rao et al. [20]	Type 1 diabetes	Plasma EVs	Proteomics, flow cytometry	Elevated EV PD‐L1 before Type 1 diabetes onset.
Diaz Lozano et al. [21]	Type 1 diabetes	Plasma‐derived EVs and whole plasma	Proteomics	Identified ∼300 β‐cell‐specific proteins in EVs; absent in whole plasma.
Melo et al. [23]	Pancreatic cancer	Tumor‐derived and serum EVs	Proteomics, flow cytometry, and immunoassay	GPC1+ EVs as promising biomarkers for early pancreatic cancer diagnosis.
Zheng et al. [24]	Colorectal cancer	Plasma EVs	Proteomics	Fibrinogen α chain‐positive EVs differentiate CRC patients from controls.
Dash et al. [25]	Colorectal cancer	Plasma EVs	Proteomics	ADAM10, CD59, TSPAN9 as markers; comparable to non‐EV‐based marker CEA.
Turay et al. [27]	Prostate cancer	Serum EVs	Proteomics	EV protein profiles reflect diagnostic differences across ethnicities.
Zhang et al. [28]	Ovarian cancer	Plasma EVs	Proteomics	EV profiles linked to diagnosis and prognosis in ovarian cancer.
Chen et al. [29]	Breast cancer	Plasma EVs	Phospho‐proteomics	Identified 144 phosphoproteins elevated in breast cancer patients.
Tian et al. [31]	Breast cancer	Plasma EVs	Proteomics	An 8‐protein signature distinguishes metastatic from non‐metastatic cases.

Biomarkers derived from EV components may also yield mechanistic insights into the associated disease. In the context of the autoimmune disease Type 1 diabetes, plasma EV composition may reflect pathophysiology, and EV cargo is associated with heterogeneity in disease progression [17]. Platelet basic protein (PPBP/CXCL7) in plasma EVs has been identified as a key regulator of pancreatic β‐cell apoptosis and macrophage activity [18, 19]. Plasma EV PD‐L1 levels are elevated in euglycemic individuals at high risk for Type 1 diabetes (islet autoantibody‐positive individuals). EV surface PD‐L1 binds directly to PD‐1 on CD8+ T cells, inhibiting their proliferation, activation, and cytotoxicity, and positively correlates with residual β‐cell function at the time of clinical diabetes onset (Table 1) [20]. Proteomics analysis in non‐obese diabetic mouse models has identified unique proteins related to β‐cell function and immune regulation in EV‐enriched plasma; importantly, over one‐third of these proteins were undetectable in whole plasma (Table 1) [21]. This integrated approach of profiling plasma‐derived EV fractions alongside whole plasma could potentially enhance the depth and detection of tissue‐specific biomarkers for autoimmune diseases, such as Type 1 diabetes.

Studies have also explored the potential for EVs as sources of cancer biomarkers. Tumor‐derived EVs carrying GPC1 have shown promise in pancreatic cancer diagnosis (Table 1) [22, 23]. Plasma‐derived EV protein analysis has identified biomarkers for several cancers, including fibrinogen α chain‐positive EVs in colorectal cancer, which can distinguish patients from healthy individuals (Table 1) [24]. Additionally, ADAM10, CD59, and TSPAN9 have been identified as early‐stage colorectal cancer biomarkers, with CD59 and TSPAN9 showing comparable diagnostic efficacy to non‐EV‐based markers like CEA (Table 1) [25]. In ovarian and prostate cancers, specific EV proteins have been linked to disease detection and monitoring, while plasma‐derived EV phosphoproteins have provided insights into breast cancer progression (Table 1) [26, 27, 28, 29, 30]. Plasma EV protein profiling has also revealed an eight‐protein signature that distinguishes metastatic from non‐metastatic breast cancer with high accuracy, aiding in treatment monitoring and survival prediction [31].

These findings collectively underscore the ability of plasma‐derived EVs to provide molecular signatures for early diagnosis and disease monitoring across diverse conditions. However, it is worth mentioning that most studies on EV‐derived biomarkers are still in the discovery phase, with few validation studies having been completed.

## 3 Challenges in the Development of Protein Biomarkers From EVs

### 3.1 Developing Biomarker Candidates From Plasma EVs Has a Few Major Challenges

#### 3.1.1 Preparation Purity

Obtaining pure EV preparations from plasma is highly complicated due to its complex matrix with the presence of proteins and particles with similar physicochemical properties to EVs. The International Society for EVs has provided guidelines to address these complexities [32, 33]. Sequential purification steps can also enhance purity but often result in substantial material losses, recovering as little as 1% of initial EVs after two rounds of purification [34, 35]. Therefore, it is impractical for biomarker studies.

#### 3.1.2 Requirement of Large Volumes of Plasma

EVs from relevant cell types, such as pancreatic β cells in Type 1 diabetes or brain‐derived EVs in Alzheimer's disease, are rare contributors to circulating EVs, which primarily originate from hematopoietic cells, including platelets and erythrocytes [29, 30, 31]. Therefore, purifying trace amounts of EVs from human plasma or serum often requires large sample volumes (up to 2 mL), which are impractical for certain populations, such as pediatric studies with limited blood draw capacities.

#### 3.1.3 The Need for Fast and Reproducible Purification Methods

To advance biomarker development through all stages, efficient and scalable sample preparation techniques are essential [21, 27, 36, 37, 38]. These techniques must be capable of accurately and specifically identifying EV protein biomarkers while ensuring high quality and reproducibility. Moreover, they should be designed to maximize cost‐effectiveness and throughput, thereby making large‐scale studies feasible. Large clinical studies often require analysis of thousands of samples. Many of the current processes involved in extracting and analyzing EV information are labor‐intensive and resource‐demanding, making them impractical for long‐term and widespread applications in EV biomarker research.

In summary, the major challenge relies on obtaining pure EV preparations from small sample sizes with purification methods that are at the same time fast, robust, and scalable.

## 4 EV Purification Methods

**FIGURE 2:**
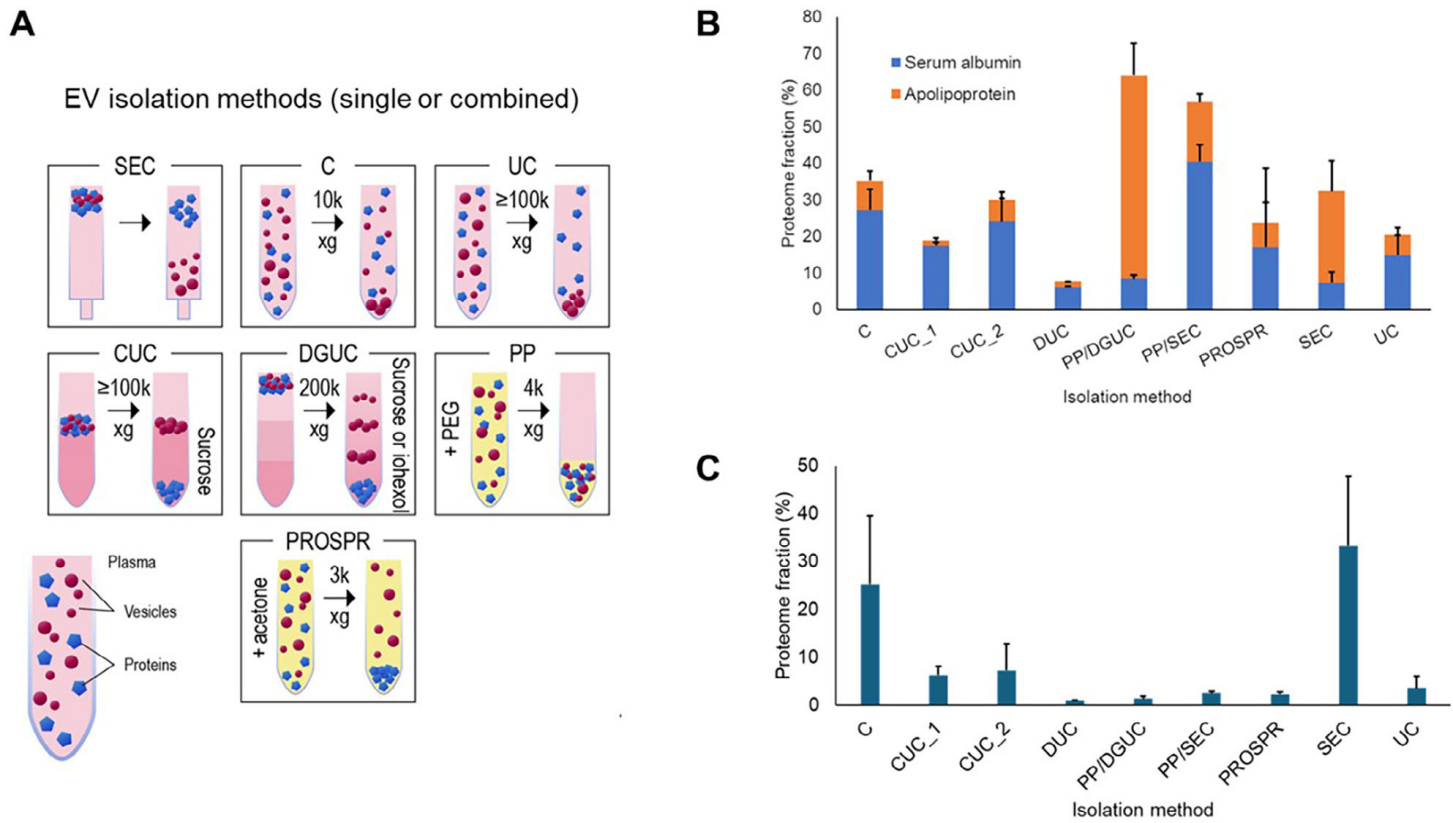
EV purification methods for proteomics analysis. (A) Different approaches are used to isolate plasma EVs. Proteomics data were downloaded from ProteomeXchange and processed with MaxQuant. (B) Abundances of common extracellular vesicle preparation contaminants across different purification methods. (C) Abundances of enriched EV proteome fractions across different purification methods. C, centrifugation at 10,000 × *g*; CUC, cushion ultracentrifugation; DGUC, density gradient ultracentrifugation; DUC, dilution followed by ultracentrifugation; PP, polymer‐based precipitation; PROSPR, PRotein Organic Solvent PRecipitation; SEC, size‐exclusion chromatography; UC, ultracentrifugation. Reproduced from Vallejo et al. [19] under the terms of the Creative Commons CC BY license.

As mentioned above, purification of EVs from biofluids represents the most challenging step for biomarker studies. Challenges are not restricted to the purity of the obtained material but also the variability, yields, and labor‐intensiveness [39]. A proteomics meta‐analysis comparing different methods has shown that centrifugation‐based approaches appear to have better success in separating lipoproteins, but often co‐pellet albumin along with EVs (Figure 2) [19]. Similarly, size‐exclusion chromatography (SEC) fails to achieve complete separation of EVs from biomolecules of comparable size, such as lipoproteins, thereby limiting the purity of isolated fractions (Figure 2) [19]. In terms of yields, centrifugation at lower speed (10,000 × *g*) and SEC led to the best recovery of the EV fraction [19]. Another comparative study on EVs isolated from plasma using different methods highlighted the variations in particle size distributions and proteome coverage, emphasizing the need for careful consideration in selecting a method that balances sample purity, yield, and proteome coverage [40]. Overall, all techniques have strengths and limitations.

### 4.1 Precipitation‐Based Purification Methods

Centrifugation, ultracentrifugation, and gradient ultracentrifugation are arguably some of the simplest and most popular methods for EV purification [39]. Centrifugation separates EVs based on their density and copurifies contaminants with similar densities. Ultracentrifugation can cause aggregation, changes in morphologies, and damages to EVs, which might lead to lower recovery yields (Table 2) [41]. Precipitation techniques can also be based on polymers or solubility in specific solvents. Polymer precipitation‐based techniques rely on the binding of EVs to insoluble polymers, such as polyethylene glycol (PEG) [42]. Protein Organic Solvent Precipitation (PROSPR) is based on the solubility of EV proteins in solvents, such as acetone [43]. These methods suffer from incomplete removal of contaminants, including non‐vesicular protein aggregates or lipoprotein particles. The use of organic solvents also compromises EV membrane integrity, leading to vesicular disruption and the release of intravesicular components, further complicating downstream analyses (Table 2).

**TABLE 2:** Overview of EV purification methods: principles, advantages, and limitations.

Method	Principles	Advantages	Limitations
Centrifugation	Separates EVs based on their density and size by spinning samples at high speeds.	Simple and easily accessible in most labs; effective at separating buoyant components like low‐density lipoproteins.	Often co‐pellet EVs with high‐density lipoproteins and other proteins. May suffer from technical variability and compromise EV integrity.
Polymer precipitation	Uses polymers to precipitate EVs out of solution based on hydrophobicity.	Cost‐effective and simple; no need for specialized equipment.	Co‐enriches for lipoproteins.
Protein organic solvent precipitation (PROSPR)	Utilizes organic solvents to selectively precipitate non‐EV proteins.	Like polymer precipitation in cost‐effectiveness and simplicity.	Disruption of EV membranes; incomplete removal of contaminants like lipoproteins.
Immunoaffinity	Targets specific EV surface proteins with antibodies immobilized on beads for selective capture.	Allows for targeting specific EV populations based on their surface markers (e.g., tetraspanins).	Low yield, complex protocols, and contamination with antibody‐binding proteins. May affect downstream analyses due to sample integrity issues.
Charge‐based method	Leverages electrostatic surface properties of EVs for isolation using solid‐phase materials.	Scalable, robust, and high purity of EV subpopulations; effective separation from plasma proteins.	Requires optimization for biological variability in EV charge profiles.
Size‐exclusion chromatography (SEC)	Separates particles based on size using a stationary porous matrix.	Simple method, leads to high recovery, and maintains EV integrity due to gentle separation.	Incomplete separation from similarly sized biomolecules, resulting in limited purity.
Asymmetrical flow field‐flow fractionation (AF4)	Utilizes a parabolic flow combined with cross flow to separate EVs based on size in an asymmetrical channel.	High‐resolution, non‐interactive separation preserving EV integrity; adaptable for various samples.	Requires precise parameter optimization (e.g., flow rates) may need adjustments for specific sample properties.
Multimodal chromatography	Uses multiple separation principles, such as charge + size.	Can be scalable and capable of effective separate EVs from plasma contaminants.	Still need further development for high‐throughput applications.

### 4.2 Immunoaffinity Purification

Membrane proteins, such as cluster of differentiation (CD) markers, on EV surfaces facilitate immunoaffinity‐based purification techniques [44]. These capture methods use antibodies or affinity ligands designed to target EV surface markers, such as tetraspanins (CD9, CD63, and CD81), for selective binding and purification [45, 46]. Although immunoaffinity methods can enrich EVs, they present challenges, including low yield, high background, and potential analytical bias resulting from EV heterogeneity. Furthermore, the robust binding between antibodies and antigens complicates the elution process, potentially damaging the EV structure and function and affecting downstream analyses (Table 2) [47]. These methods often require complex protocols, limiting their broader applicability.

### 4.3 Charge‐Based Purification Methods

These methods leverage EV's inherent electrostatic surface properties, mostly characterized by negative lipid components like phosphatidylserine [48]. Therefore, charge‐based purifications are usually performed by anion exchange chromatography and have the advantage of being fast and scalable (Table 2) [49, 50]. However, a key challenge is managing biological variability among samples, which influences the EV surface charge and affects purification efficiency. The Mag‐Net method is a recently introduced technique for purifying EVs from plasma, utilizing hyper‐porous strong‐anion exchange (SAX) magnetic microparticles to sieve membrane‐bound particles based on size and charge [51]. Mag‐Net is robust, inexpensive, and requires small plasma input (∼100 µL or less), making it suitable for high‐throughput applications with automated processing capabilities [52]. The technique still requires precise optimization for various sample types and research objectives to ensure scalability and reproducibility in diverse studies.

### 4.4 Size‐Based Techniques

SEC is among the most popular techniques for EV purification. SEC is a gentle technique, helping to preserve the EV morphology and leading to high yields of recovery [19, 53]. The technique is remarkably robust and can be performed with small plasma volumes (∼50 µL). Another advantage is that the technique is fast and fully automatable [54]. However, SEC copurifies contaminants of similar size, such as lipoproteins and protein aggregates (Table 2) [19]. Asymmetrical flow field‐flow fractionation (AF4) is an advanced size‐based technique for separating EVs based on their size through a non‐invasive process that preserves sample integrity. Unlike SEC, which relies on the pore size of the resin, AF4 employs an asymmetrical channel design featuring an impermeable upper block and a lower block with a semipermeable membrane [55]. In AF4, a parabolic carrier flow transports the sample, while a perpendicular cross flow pushes particles toward the membrane [56]. Smaller particles diffuse rapidly and elute earlier as they are carried further from the membrane's influence, whereas larger particles remain closer to the membrane and elute later [57]. The unique flow dynamics in AF4 enable high‐resolution, non‐interactive separation while maintaining EV integrity. Coupled with detectors such as multi‐angle light scattering (MALS) and UV, AF4 provides precise EV characterization, making it a powerful tool for analyzing complex biological samples. Despite its benefits, AF4 faces challenges such as the need for precise optimization of parameters like flow rates and ultrafiltration conditions to prevent EV loss (Table 2) [57]. Ultrafiltration is another size‐based technique for EV purification, which consists of passing the samples through a small pore filter. Ultrafiltration is a little difficult to apply to the plasma samples due to their viscosity and small volumes. Therefore, they are often used in combination with other techniques to help concentrate samples, such as in the case of AF4 mentioned above [57].

### 4.5 Multimodal Chromatography‐Based Methods

These methods have the advantage of purifying EVs by multiple physicochemical properties without the need for sequential steps and their associated sample losses. A combined SEC‐cation exchange column can drastically reduce lipoprotein contamination from plasma purification without the extreme sample losses of sequential purifications [34, 35, 58]. Another example is the multimodal flow‐through chromatography. In this chromatography, the resin with size‐selective pores absorbs the contaminant, while EVs are unable to be absorbed by these pores and are collected in the flow‐through. A multimodal flow‐through chromatography using an anion exchange column was tested with plasma samples, leading to a high yield of EV recovery, but was unable to deplete lipoproteins (Table 2) [37]. Multimodal chromatography‐based methods have potential as alternative approaches to purify EVs, but they still need further development to become routine methods for biomarker applications.

Overall, all methods have their advantages and disadvantages with sequential purification steps required to improve EV purity at the cost of compromised sample recovery.

## 5 Refining the EV Composition

As mentioned above, no single method can purify EVs to homogeneity [59]. Obtaining homogeneous purity may not be feasible even when employing a combination of methods [59], but they can be used to at least refine the EV composition. Hence, the question remains whether pure EV populations are necessary or if mixed populations can still yield reliable biomarker information [60]. We believe that a more viable alternative is to better understand the composition of EVs and utilize proteomics to quantify EV‐specific proteins in enriched, yet still complex, fractions. This will enable maximizing EV recovery from small sample volumes in a scalable fashion [54].

**FIGURE 3:**
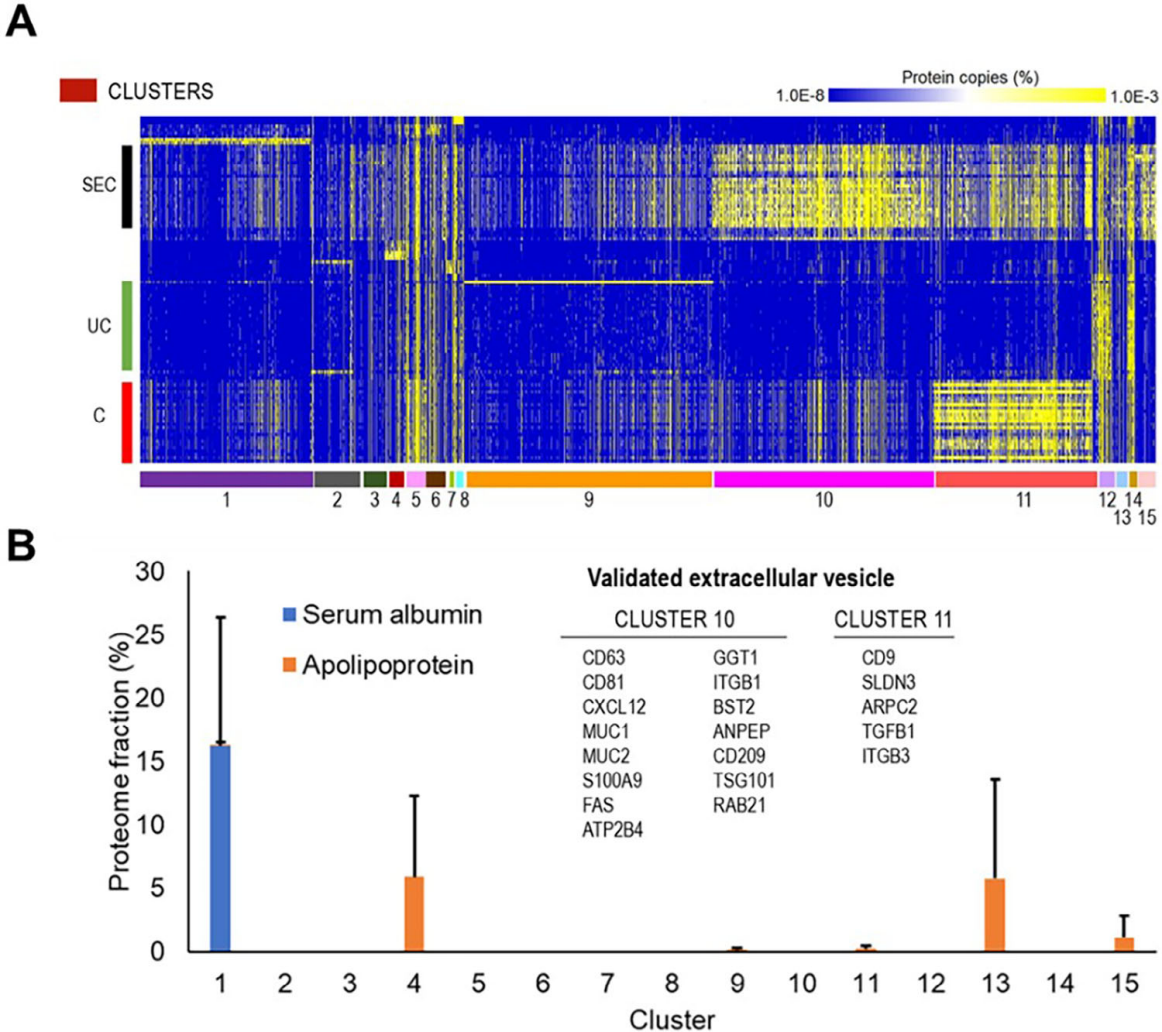
Clusters of the plasma EV proteome. Proteomics data from plasma EVs purified with the methods mentioned in Figure 2 underwent clustering analysis. (A) The highest enriched clusters with the top 100 EV proteins from Vesiclepedia. C, centrifugation at 10,000 × *g*; EV, extracellular vesicle; SEC, size‐exclusion chromatography; UC, ultracentrifugation. (B) Abundances of common extracellular vesicle preparation contaminants across different clusters of the proteome meta‐analysis. Embedded is the list of validated classical EV markers enriched in Clusters 10 and 11. Reproduced from Vallejo et al. [19] under the terms of the Creative Commons CC BY license.

We recently developed a meta‐analysis approach to refine the composition of plasma EVs [19]. This approach is based on the concept that each purification method will yield different contaminant‐to‐EV ratios, resulting in distinct abundance profiles between contaminants and EV proteins. When the abundance profiles are clustered, EV proteins cluster with EV markers, while contaminants are separated into distinct clusters (Figure 3). We validated this approach by calculating true positive rates based on EV proteins previously confirmed by immunogold electron microscopy, such as CD9, CD63, and CD81 [61, 62, 63]. We found that SEC resulted in the best contaminant‐to‐EV ratios while maintaining EV characteristics. By integrating multiple advanced methods, it is possible to map out the EV compositional landscape independent of sample purity.

Another way to refine EV composition is to validate specific proteins by image‐based methods, such as immunogold transmission electron microscopy and ExoView technology. In immunogold analysis, EV proteins are labeled with specific antibodies conjugated with gold particles, which are visualized by transmission electron microscopy [64]. With ExoView technology, EVs are captured in micro‐chips using anti‐CD9, ‐CD63, and ‐CD81 antibodies, then proteins are detected by immunofluorescence using specific antibodies [65]. We have recently used ExoView to validate the presence of platelet basic protein in plasma EVs [19]. Unfortunately, neither of these approaches is high throughput as only specified proteins can be validated at a time.

## 6 MS in EV Analysis

**FIGURE 4:**
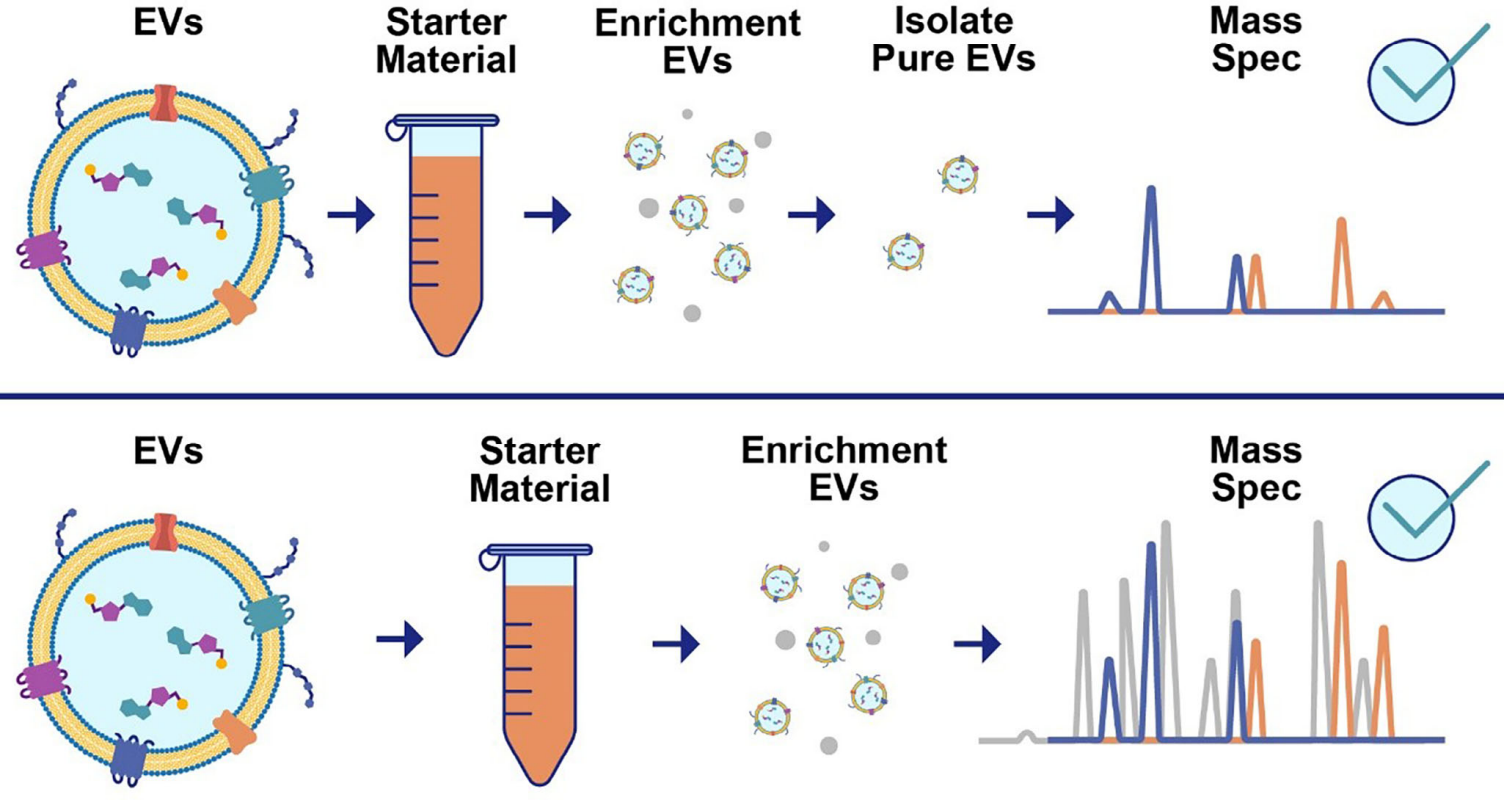
Perspective on MS‐based extracellular vesicle (EV) proteomics in enriched yet complex samples.

The high capacity of modern MS provides a window of opportunity to obtain reliable biological information from EVs. MS has gained prominence in the examination of EV fractions, significantly contributing to biomarker discovery efforts [66]. With ongoing advancements in MS instrumentation, coupled with improvements in acquisition methods, researchers can achieve deep coverage, high sensitivity, and robust reproducibility in shorter time frames [67, 68]. This allows for the analysis of EVs with complex contaminant backgrounds without compromising the coverage and quantification of EV proteins. The large sample losses associated with sequential purification steps may have a greater impact on EV proteome coverage compared to analysis on enriched but still complex samples (Figure 4).

### 6.1 Untargeted Proteomics: Data‐Independent Acquisition (DIA) and Data‐Dependent Acquisition (DDA)

DIA and DDA are excellent approaches for identifying EV proteins even within complex samples. Both approaches can identify and quantify over 10,000 proteins from a single sample, providing deep coverage of the EV proteome even when contaminants are present. The conceptual difference is that in DDA, the most abundant precursor ions are selected for isolation and fragmentation, and the monitoring of all product ions is performed. With signal intensity as the primary selection criterion, the more abundant species are favored for fragmentation [69]. DIA is increasingly becoming the preferred method for proteomics analysis because it helps to overcome the bias (i.e., under‐sampling) against low‐abundant ions. Therefore, this leads to a better quantification of the samples. For example, Zheng et al. have used both DDA and DIA techniques to study EV components as colorectal cancer biomarkers. They performed a discovery phase experiment using tandem‐mass tags labeling and DDA followed by a validation experiment with DIA, leading to the identification of biomarker candidates with area under the curve (AUC) of up to 1.0 [24]. Therefore, demonstrating the potential of these approaches for biomarker studies.

### 6.2 Targeted Proteomics—Selected Reaction Monitoring (SRM)

Once the EV composition is known, targeted proteomics is an excellent alternative for quantifying their proteins, even in complex samples. SRM analysis is carried out on triple quadrupole (QQQ) and quadrupole‐trap (QTrap) mass spectrometer platforms, where only pre‐identified peptides are measured, thereby filtering out signals from contaminant proteins. In addition, isotope‐labeled surrogate peptides are spiked into the samples, ensuring the identity of the peptide and providing reliable quantification [70, 71, 72]. For instance, SRM has been used to validate changes in the abundance of prostate cancer biomarker candidates in urinary EVs [73]. In addition to SRM, parallel reaction monitoring (PRM) is an acquisition mode that leverages the technical advancements of the quadrupole time‐of‐flight (Q‐TOF) and Orbitrap series instruments, enabling high‐resolution monitoring of target peptides [74, 75, 76]. In biomarker studies, PRM has been used to validate candidates for early prognosis of acute respiratory distress syndrome, leading to the identification of biomarker panels with an AUC up to 0.802 [77]. Compared to untargeted proteomics, targeted proteomics offers a more reliable and sensitive quantification of EV proteins. However, targeted proteomics can only measure several hundred peptides at a time, compared to tens of thousands of peptides that can be measured by untargeted proteomics.

### 6.3 Hybrid DIA‐PRM

In ongoing efforts to streamline discovery and quantitative proteomics, a combination of DIA and PRM acquisition methods has recently emerged as a promising approach. In a recent publication, the hybrid‐DIA acquisition strategy described the use of an Application Programming Interface (API) to dynamically intercalate DIA scans with multiplexed tandem MS scans of predefined peptide targets, for which stable isotope‐labeled standards were spiked in [78]. Conceptually, the hybrid‐DIA MS acquisition strategy is promising, as it combines unbiased DIA‐based profiling with hypothesis‐driven quantification, while also offering the advantages of increased throughput and coverage in a single run. Nevertheless, more work is needed to determine the reach of this technology and to develop software for data integration.

## 7 Machine Learning to Determine Biomarker Performance and Panels

Machine learning (ML) tools are crucial for improving the analysis of large datasets. Unlike traditional approaches with pre‐specified models, ML approaches use algorithms to directly learn a model from the data [79], being particularly attractive for the highly complex biomolecular composition of EVs [4, 80]. ML approaches require greater volumes of data (relative to traditional methods) to yield adequate models, which can be a significant challenge for certain biomedical applications. However, achieving high volumes of data has become increasingly feasible due to advancements in the high‐throughput technologies described above. For instance, a hybrid ML algorithm based on three approaches—LsBoost, convolutional neural networks, and support vector machines (SVMs)—on a cohort of 100 breast cancer patients versus 30 controls identified three EV protein biomarkers whose modeled signature exhibited 100% sensitivity and 80% specificity in detecting triple‐negative breast cancer, a breast cancer subtype void of therapeutic and diagnostic targets [81]. ML is especially strong in selecting important features in the data, which can lead to the identification of the best biomarker candidates. Bukva et al. used the Least Absolute Shrinkage and Selection Operator (LASSO) method to quantify the importance of various proteins in discriminating between the EVs from 60 cell lines comprising of nine different tumor types (breast, central nervous system, colon, kidney, leukemia, lung, melanoma, ovary, and prostate). Contrasting with the classification performance of the model based on the entire proteome (accuracy = 49.15%), the model based on the subset of LASSO‐selected proteins achieved 91.68% accuracy in discriminating between the EVs across nine tumor types [82].

In summary, ML has not only shown predictive efficacy of EV‐based models but also facilitates in the identification of critical biomarkers that distinguish their composition, driving advancements in EV research and diagnostics.

## 8 Future Directions

In EV research, the identification of novel protein biomarkers holds great promise for advancing diagnostic and therapeutic applications. Although existing workflows have often emphasized the need for highly purified EV fractions to ensure accuracy in biomarker discovery, we propose that this stringency may not be necessary. Technologies like MS enable the analysis of EV markers within complex biological mixtures, thereby accelerating the discovery process and facilitating higher‐throughput screenings. Nevertheless, comprehensive characterization and validation of the EV composition remain the most urgent need in the field. As mentioned above, we recently developed a meta‐analysis method based on protein clustering to refine the plasma EV proteome. We believe that ML might also be able to contribute to refining the EV composition. A current bottleneck is the lack of an image‐based, high‐throughput method for validating protein localization to vesicles. Immunogold transmission electron microscopy and ExoView are excellent technologies; however, the development of a highly multiplexed single‐EV imaging technology would greatly benefit the field. In summary, the success of this approach relies on having validated EV proteins to be quantified by MS. Therefore, we believe that this should be a focus for the coming years.

In terms of purification methods, though improving purity is always desirable, we believe the focus should shift toward increasing recovery from smaller sample sizes. Methods, such as SEC and Mag‐Net, which require less than 100 µL, have great potential. Furthermore, these methods are automatable, allowing for the consistent preparation of hundreds to thousands of samples. Microfluidic devices have been used to isolate EVs [83]. Microfluidic systems, such as digital EV screening (DEST), present a promising alternative to addressing these sensitivity challenges, facilitating high‐throughput, multiplexed analysis of individual EVs [84]. A major challenge in microfluidics remains the recovery of sufficient material for proteomics analysis. However, the sensitivity of the mass spectrometers has been continuously improving, making an impact on the field of single‐cell proteomics. The powerful tools developed in this field could contribute to the precise measurement of EVs using specialized processing platforms like the Nanodroplet Processing in One pot for Trace Samples (NanoPOTS) [85].

As MS instrumentation continues to advance, it will allow for deeper coverages of the EV proteome in shorter analysis times. For instance, the Thermo Astral mass spectrometer can identify and quantify thousands of proteins with chromatography gradients that are a few minutes long [86]. This would generate massive amounts of data that conventional statistical analysis by itself might not be able to extract all the important information. In this context, ML can make a significant contribution to identifying the most effective biomarkers or panels of biomarkers.

Regarding the continued development of current biomarker candidates, rigorous validation steps are crucial for transitioning them from the bench to the bedside. These validation approaches are necessary to confirm the specificity, sensitivity, and functional relevance of identified biomarkers, ensuring statistical significance and biological meaning within disease contexts. Validation tests ensure reliable detection and quantification of biomarkers across different populations, ensuring the performance and reproducibility of the assay.

## 9 Conclusions

Obtaining pure EV preparations poses a significant challenge to the development of biomarkers based on EV components. Here, we explored the concept that pure EV preparations might not be viable for biomarker studies due to the need for large sample volumes and multiple purification steps. Therefore, a better characterization of the EV composition, followed by deep proteomics analysis in enriched but still complex samples, might be a more viable alternative.

## Conflicts of Interest

The authors declare no conflicts of interest.

## Data Availability

The authors have nothing to report.

## References

[1] E. I. Buzas , “The Roles of Extracellular Vesicles in the Immune System,” Nature Reviews Immunology 23 (2023): 236–250. 10.1038/s41577-022-00763-8 PMC9361922 35927511

[2] E. Woith , G. Fuhrmann , and M. F. Melzig , “Extracellular Vesicles‐Connecting Kingdoms,” International Journal of Molecular Sciences 20 (2019): 5695. 31739393 10.3390/ijms20225695 PMC6888613

[3] M. C. Ciferri , R. Quarto , and R. Tasso , “Extracellular Vesicles‐Connecting Kingdoms,” International Journal of Molecular Sciences 10 (2021): 100255.

[4] V. Bettio , E. Mazzucco , A. Antona , et al., “Extracellular Vesicles From Human Plasma for Biomarkers Discovery: Impact of Anticoagulants and Isolation Techniques,” PLoS ONE 18 (2023): 0285440. 10.1371/journal.pone.0285440 PMC10171685 37163560

[5] T. Tsering , A. Nadeau , T. Wu , K. Dickinson , and J. V. Burnier , “Extracellular Vesicle‐Associated DNA: Ten Years Since Its Discovery in Human Blood,” Cell Death & Disease 15 (2024): 668. 39266560 10.1038/s41419-024-07003-y PMC11393322

[6] M. A. Kumar , S. K. Baba , H. Q. Sadida , et al., “Extracellular Vesicles as Tools and Targets in Therapy for Diseases,” Signal Transduction and Targeted Therapy 9 (2024): 27. 38311623 10.1038/s41392-024-01735-1 PMC10838959

[7] R. S. Aguirre , A. Kulkarni , M. W. Becker , et al., “Extracellular Vesicles in Beta Cell Biology: Role of Lipids in Vesicle Biogenesis, Cargo, and Intercellular Signaling,” Molecular Metabolism 2022, 63, 101545. 35817393 10.1016/j.molmet.2022.101545 PMC9294332

[8] N. Cortes‐Serra , M. T. Mendes , C. Mazagatos , et al., “Plasma‐Derived Extracellular Vesicles as Potential Biomarkers in Heart Transplant Patient With Chronic Chagas Disease,” Emerging Infectious Diseases 26 (2020): 1846–1851. 32687028 10.3201/eid2608.191042 PMC7392439

[9] R. P. Madeira , P. Meneghetti , N. Lozano , et al., “Exploring Peripheral Blood‐Derived Extracellular Vesicles As Biomarkers: Implications for Chronic Chagas Disease With Viral Infection or Transplantation,” Microorganisms 12 (2024): 116. 38257943 10.3390/microorganisms12010116 PMC10818975

[10] Y. Choi , J. H. Park , A. Jo , et al., “Blood‐Derived APLP1 + Extracellular Vesicles Are Potential Biomarkers for the Early Diagnosis of Brain Diseases,” Science Advances 11 (2025): ado6894. 10.1126/sciadv.ado6894 PMC11691634 39742488

[11] S. Yan , W. Zhang , X. Li , et al., “Single Extracellular Vesicle Detection Assay Identifies Membrane‐Associated α‐Synuclein as an Early‐Stage Biomarker in Parkinson's Disease,” Cell Reports Medicine 6 (2025): 101999. 40056910 10.1016/j.xcrm.2025.101999 PMC11970385

[12] A. Stuendl , T. Kraus , M. Chatterjee , et al., “α‐Synuclein in Plasma‐Derived Extracellular Vesicles Is a Potential Biomarker of Parkinson's Disease,” Movement Disorders 36 (2021): 2508–2518. 34002893 10.1002/mds.28639

[13] S. Yan , C. Jiang , A. Janzen , et al., “Neuronally Derived Extracellular Vesicle α‐Synuclein as a Serum Biomarker for Individuals at Risk of Developing Parkinson Disease,” JAMA Neurology 81 (2024): 59. 38048087 10.1001/jamaneurol.2023.4398 PMC10696516

[14] T. Gilboa , D. Ter‐Ovanesyan , S.‐C. Wang , et al., “Measurement of α‐Synuclein as Protein Cargo in Plasma Extracellular Vesicles,” Proceedings of the National Academy of Sciences of the United States of America 121 (2024): 2408949121. 10.1073/pnas.2408949121 PMC11551346 39475636

[15] E. Vacchi , J. Burrello , D. Di Silvestre , et al., “Immune Profiling of Plasma‐Derived Extracellular Vesicles Identifies Parkinson Disease,” Neurology Neuroimmunology & Neuroinflammation 7 (2020): 866. 10.1212/NXI.0000000000000866 PMC7428368 32817412

[16] M. Chatterjee , S. Özdemir , C. Fritz , et al., “Plasma Extracellular Vesicle Tau and TDP‐43 as Diagnostic Biomarkers in FTD and ALS,” Nature Medicine 30 (2024): 1771–1783. 10.1038/s41591-024-02937-4 PMC11186765 38890531

[17] C. N. Suire and M. D. Hade , “Extracellular Vesicles in Type 1 Diabetes: A Versatile Tool,” Bioengineering (Basel) 9 (2022): 105. 35324794 10.3390/bioengineering9030105 PMC8945706

[18] E. S. Nakayasu , L. M. Bramer , C. Ansong , et al., “Plasma Protein Biomarkers Predict the Development of Persistent Autoantibodies and Type 1 Diabetes 6 Months Prior to the Onset of Autoimmunity,” Cell Reports Medicine 4 (2023): 101093. 37390828 10.1016/j.xcrm.2023.101093 PMC10394168

[19] M. C. Vallejo , S. Sarkar , E. C. Elliott , et al., “A Proteomic Meta‐Analysis Refinement of Plasma Extracellular Vesicles,” Scientific Data 10 (2023): 837. 38017024 10.1038/s41597-023-02748-1 PMC10684639

[20] C. Rao , D. T. Cater , S. Roy , et al., “Beta Cell Extracellular Vesicle PD‐L1 as a Novel Regulator of CD8+ T Cell Activity and Biomarker During the Evolution of Type 1 Diabetes,” Diabetologia 68 (2025): 382–396. 39508879 10.1007/s00125-024-06313-2 PMC12276980

[21] I. M. Diaz Lozano , H. Sork , V. M. Stone , et al., “Proteome Profiling of Whole Plasma and Plasma‐Derived Extracellular Vesicles Facilitates the Detection of Tissue Biomarkers in the Non‐Obese Diabetic Mouse,” Frontiers in Endocrinology (Lausanne) 13 (2022): 971313. 10.3389/fendo.2022.971313 PMC9563222 36246930

[22] F. Lucien , V. Lac , D. D. Billadeau , A. Borgida , S. Gallinger , and H. S. Leong , “Glypican‐1 and Glycoprotein 2 Bearing Extracellular Vesicles Do Not Discern Pancreatic Cancer From Benign Pancreatic Diseases,” Oncotarget 10 (2019): 1045–1055. 30800217 10.18632/oncotarget.26620 PMC6383691

[23] S. A. Melo , L. B. Luecke , C. Kahlert , et al., “Glypican‐1 Identifies Cancer Exosomes and Detects Early Pancreatic Cancer,” Nature 523 (2015): 177–182. 26106858 10.1038/nature14581 PMC4825698

[24] X. Zheng , K. Xu , B. Zhou , et al., “A Circulating Extracellular Vesicles‐Based Novel Screening Tool for Colorectal Cancer Revealed by Shotgun and Data‐Independent Acquisition Mass Spectrometry,” Journal of Extracellular Vesicles 9 (2020): 1750202. 32363013 10.1080/20013078.2020.1750202 PMC7178829

[25] S. Dash , C. C. Wu , C. C. Wu , et al., “Extracellular Vesicle Membrane Protein Profiling and Targeted Mass Spectrometry Unveil CD59 and Tetraspanin 9 As Novel Plasma Biomarkers for Detection of Colorectal Cancer,” Cancers (Basel) 15 2022, 177. 36612172 10.3390/cancers15010177 PMC9818822

[26] T. S. Worst , J. Von Hardenberg , J. C. Gross , et al., “Database‐Augmented Mass Spectrometry Analysis of Exosomes Identifies Claudin 3 as a Putative Prostate Cancer Biomarker,” Molecular & Cellular Proteomics 16 (2017): 998–1008. 28396511 10.1074/mcp.M117.068577 PMC5461549

[27] D. Turay , S. Khan , C. J. Diaz Osterman , et al., “Proteomic Profiling of Serum‐Derived Exosomes From Ethnically Diverse Prostate Cancer Patients,” Cancer Investigation 34 (2016): 1–11. 26536157 10.3109/07357907.2015.1081921 PMC4732892

[28] W. Zhang , X. Ou , and X. Wu , “Proteomics Profiling of Plasma Exosomes in Epithelial Ovarian Cancer: A Potential Role in the Coagulation Cascade, Diagnosis and Prognosis,” International Journal of Oncology 54 (2019): 1719–1733. 30864689 10.3892/ijo.2019.4742 PMC6438431

[29] I.‐H. Chen , L. Xue , C.‐C. Hsu , et al., “Phosphoproteins in Extracellular Vesicles as Candidate Markers for Breast Cancer,” Proceedings of the National Academy of Sciences of the United States of America 114 (2017): 3175–3180. 28270605 10.1073/pnas.1618088114 PMC5373352

[30] E. Manouchehri Doulabi , C. Fredolini , R. Gallini , et al., “Surface Protein Profiling of Prostate‐Derived Extracellular Vesicles by Mass Spectrometry and Proximity Assays,” Communications Biology 5 (2022): 1402. 36550367 10.1038/s42003-022-04349-x PMC9780212

[31] F. Tian , S. Zhang , C. Liu , et al., “Protein Analysis of Extracellular Vesicles to Monitor and Predict Therapeutic Response in Metastatic Breast Cancer,” Nature Communications 12 (2021): 2536. 10.1038/s41467-021-22913-7 PMC8100127 33953198

[32] C. Théry , K. W. Witwer , E. Aikawa , et al., “Minimal Information for Studies of Extracellular Vesicles 2018 (MISEV2018): A Position Statement of the International Society for Extracellular Vesicles and Update of the MISEV2014 Guidelines,” Journal of Extracellular Vesicles 7 (2018): 1535750. 30637094 10.1080/20013078.2018.1535750 PMC6322352

[33] J. A. Welsh , D. C. I. Goberdhan , L. O'driscoll , et al., “Minimal Information for Studies of Extracellular Vesicles (MISEV2023): From Basic to Advanced Approaches,” Journal of Extracellular Vesicles 13 (2024): 12404. 10.1002/jev2.12404 PMC10850029 38326288

[34] R. Xu , D. W. Greening , A. Rai , H. Ji , and R. J. Simpson , “Highly‐Purified Exosomes and Shed Microvesicles Isolated From the Human Colon Cancer Cell Line LIM1863 by Sequential Centrifugal Ultrafiltration Are Biochemically and Functionally Distinct,” Methods (San Diego, Calif.) 87 (2015): 11–25. 25890246 10.1016/j.ymeth.2015.04.008

[35] G. Vergauwen , J. Tulkens , C. Pinheiro , et al., “Robust Sequential Biophysical Fractionation of Blood Plasma to Study Variations in the Biomolecular Landscape of Systemically Circulating Extracellular Vesicles Across Clinical Conditions,” Journal of Extracellular Vesicles 10 (2021): 12122. 10.1002/jev2.12122 PMC8363909 34429857

[36] D. Ter‐Ovanesyan , T. Gilboa , B. Budnik , et al., “Improved Isolation of Extracellular Vesicles By Removal of Both Free Proteins and Lipoproteins,” eLife 12 2023, e86394. 10.7554/eLife.86394 PMC10229111 37252755

[37] S. E. Bonner , S. I. Van De Wakker , W. Phillips , et al., “Scalable Purification of Extracellular Vesicles With High Yield and Purity Using Multimodal Flowthrough Chromatography,” Journal of Extracellular Biology 3 (2024): 138. 10.1002/jex2.138 PMC11080796 38939900

[38] M. Smolarz , M. Pietrowska , N. Matysiak , L. Mielanczyk , and P. Widlak , “Proteome Profiling of Exosomes Purified from a Small Amount of Human Serum: The Problem of Co‐Purified Serum Components,” Proteomes 7 (2019): 18. 31035355 10.3390/proteomes7020018 PMC6630217

[39] M. Clos‐Sansalvador , M. Monguió‐Tortajada , S. Roura , M. Franquesa , and F. E. Borràs , “Commonly used Methods for Extracellular Vesicles′ Enrichment: Implications in Downstream Analyses and Use,” European Journal of Cell Biology 101 (2022): 151227. 35460958 10.1016/j.ejcb.2022.151227

[40] P. S. Suresh and Q. Zhang , “Comprehensive Comparison of Methods for Isolation of Extracellular Vesicles From Human Plasma,” Journal of Proteome Research 24 (2025): 2956–2967. 40356199 10.1021/acs.jproteome.5c00149 PMC12150312

[41] R. Linares , S. Tan , C. Gounou , N. Arraud , and A. R. Brisson , “High‐Speed Centrifugation Induces Aggregation of Extracellular Vesicles,” Journal of Extracellular Vesicles 4 (2015): 29509. 26700615 10.3402/jev.v4.29509 PMC4689953

[42] M. A. Rider , S. N. Hurwitz , and D. G. Meckes Jr , “ExtraPEG: A Polyethylene Glycol‐Based Method for Enrichment of Extracellular Vesicles,” Scientific Reports 6 (2016): 23978. 27068479 10.1038/srep23978 PMC4828635

[43] X. Gallart‐Palau , A. Serra , A. S. W. Wong , et al., “Extracellular Vesicles Are Rapidly Purified From human Plasma by PRotein Organic Solvent PRecipitation (PROSPR),” Scientific Reports 5 (2015): 14664. 26419333 10.1038/srep14664 PMC4588595

[44] N. Kastelowitz and H. Yin , “Exosomes and Microvesicles: Identification and Targeting by Particle Size and Lipid Chemical Probes,” ChemBioChem 15 (2014): 923–928. 24740901 10.1002/cbic.201400043 PMC4098878

[45] N. Zarovni , A. Corrado , P. Guazzi , et al., “Integrated Isolation and Quantitative Analysis of Exosome Shuttled Proteins and Nucleic Acids Using Immunocapture Approaches,” Methods (San Diego, Calif.) 87 (2015): 46–58. 26044649 10.1016/j.ymeth.2015.05.028

[46] Z. Andreu and M. Yáñez‐Mó , “Tetraspanins in Extracellular Vesicle Formation and Function,” Frontiers in Immunology 5 (2014): 442. 25278937 10.3389/fimmu.2014.00442 PMC4165315

[47] W. Su , H. Li , W. Chen , and J. Qin , “Microfluidic Strategies for Label‐Free Exosomes Isolation and Analysis,” TrAC‐Trends in Analytical Chemistry 118 (2019): 686–698.

[48] W. Back , M. Bang , J.‐H. Jung , et al., “Charge‐Based Isolation of Extracellular Vesicles From Human Plasma,” ACS Omega 9 (2024): 17832–17838. 38680311 10.1021/acsomega.3c07427 PMC11044138

[49] N. Heath , L. Grant , T. M. De Oliveira , et al., “Rapid Isolation and Enrichment of Extracellular Vesicle Preparations Using Anion Exchange Chromatography,” Scientific Reports 8 (2018): 5730. 29636530 10.1038/s41598-018-24163-y PMC5893571

[50] X. Su , G. P. O. Júnior , A.‐L. Marie , et al., “Enhanced Proteomic Profiling of Human Plasma‐Derived Extracellular Vesicles Through Charge‐Based Fractionation to Advance Biomarker Discovery Potential,” Journal of Extracellular Vesicles 13 (2024): 70024. 10.1002/jev2.70024 PMC11621968 39641316

[51] C. C. Wu , K. A. Tsantilas , J. Park , et al., “Enrichment of Extracellular Vesicles Using Mag‐Net for the Analysisof the Plasma Proteome,” Nature Communications 16 (2025): 5447. 10.1038/s41467-025-60595-7 PMC12219689 40595564

[52] A. Iliuk , X. Wu , L. Li , et al., “Plasma‐Derived Extracellular Vesicle Phosphoproteomics Through Chemical Affinity Purification,” Journal of Proteome Research 19 (2020): 2563–2574. 32396726 10.1021/acs.jproteome.0c00151 PMC7479851

[53] A. Gamez‐Valero , M. Monguio‐Tortajada , L. Carreras‐Planella , et al., “Size‐Exclusion Chromatography‐Based Isolation Minimally Alters Extracellular Vesicles' Characteristics Compared to Precipitating Agents,” Scientific Reports 2016, 6, 33641. 27640641 10.1038/srep33641 PMC5027519

[54] I. Díaz Ludovico , S. M. Powell , G. Many , et al., “A Fast and Sensitive Size‐Exclusion Chromatography Method for Plasma Extracellular Vesicle Proteomic Analysis,” Proteomics 24 (2024): 2400025. 10.1002/pmic.202400025 PMC11610398 38895962

[55] D. Kang , S. Oh , S.‐M. Ahn , B.‐H. Lee , and M. H. Moon , “Proteomic Analysis of Exosomes From Human Neural Stem Cells by Flow Field‐Flow Fractionation and Nanoflow Liquid Chromatography−Tandem Mass Spectrometry,” Journal of Proteome Research 7 (2008): 3475–3480. 18570454 10.1021/pr800225z

[56] M. Baalousha , B. Stolpe , and J. R. Lead , “Flow Field‐Flow Fractionation for the Analysis and Characterization of Natural Colloids and Manufactured Nanoparticles in Environmental Systems: A Critical Review,” Journal of Chromatography 1218 (2011): 4078–4103. 21621214 10.1016/j.chroma.2011.04.063

[57] K. Agarwal , M. Saji , S. M. Lazaroff , A. F. Palmer , M. D. Ringel , and M. E. Paulaitis , “Analysis of Exosome Release as a Cellular Response to MAPK Pathway Inhibition,” Langmuir 31 (2015): 5440–5448. 25915504 10.1021/acs.langmuir.5b00095 PMC4589192

[58] J. Van Deun , A. Jo , H. Li , et al., “Integrated Dual‐Mode Chromatography to Enrich Extracellular Vesicles From Plasma,” Advanced Biosystem 4 (2020): 1900310. 10.1002/adbi.201900310 PMC7606548 32351054

[59] A. Hendrix , L. Lippens , C. Pinheiro , et al., “Extracellular Vesicle Analysis,” Nature Reviews Methods Primers 3 (2023): 56. 10.1038/s43586-023-00240-z PMC12968065 41809514

[60] J. D. Spitzberg , S. Ferguson , K. S. Yang , H. M. Peterson , J. C. T. Carlson , and R. Weissleder , “Multiplexed Analysis of EV Reveals Specific Biomarker Composition With Diagnostic Impact,” Nature Communications 14 (2023): 1239. 10.1038/s41467-023-36932-z PMC9985597 36870999

[61] V. Cepeda , M. Esteban , and A. Fraile‐Ramos , “Human Cytomegalovirus Final Envelopment on Membranes Containing both Trans‐Golgi Network and Endosomal Markers,” Cellular Microbiology 12 (2010): 386–404. 19888988 10.1111/j.1462-5822.2009.01405.x

[62] B. Hosseinkhani , N. Van Den Akker , J. D'haen , et al., “Direct Detection of Nano‐Scale Extracellular Vesicles Derived From Inflammation‐Triggered Endothelial Cells Using Surface Plasmon Resonance,” Nanomedicine 13 (2017): 1663–1671. 28366819 10.1016/j.nano.2017.03.010

[63] T. Pisitkun , R.‐F. Shen , and M. A. Knepper , “Identification and Proteomic Profiling of Exosomes in Human Urine,” Proceedings of the National Academy of Sciences of the United States of America 101 (2004): 13368–13373. 15326289 10.1073/pnas.0403453101 PMC516573

[64] S. T.‐Y. Chuo , J. C.‐Y. Chien , and C. P.‐K. Lai , “Imaging Extracellular Vesicles: Current and Emerging Methods,” Journal of Biomedical Science 25 (2018): 91. 30580764 10.1186/s12929-018-0494-5 PMC6304785

[65] J. M. J. Price , Y. Hisada , J. Hazeldine , et al., “Detection of Tissue Factor–Positive Extracellular Vesicles Using the ExoView R100 System,” Research and Practice in Thrombosis and Haemostasis 7 (2023): 100177. 37333992 10.1016/j.rpth.2023.100177 PMC10276261

[66] I. Jalaludin , D. M. Lubman , and J. Kim , “A Guide to Mass Spectrometric Analysis of Extracellular Vesicle Proteins for Biomarker Discovery,” Mass Spectrometry Reviews 42 (2023): 844–872. 34747512 10.1002/mas.21749

[67] Y. Jiang , D. A. B. Rex , D. Schuster , et al., “Comprehensive Overview of Bottom‐Up Proteomics Using Mass Spectrometry,” ACS Measurement Science Au 4 (2024): 338–417. 39193565 10.1021/acsmeasuresciau.3c00068 PMC11348894

[68] T. M. Peters‐Clarke , J. J. Coon , and N. M. Riley , “Instrumentation at the Leading Edge of Proteomics,” Analytical Chemistry 96 (2024): 7976–8010. 38738990 10.1021/acs.analchem.3c04497 PMC11996003

[69] Y. Zhang , B. R. Fonslow , B. Shan , M.‐C. Baek , and J. R. Yates , “Protein Analysis by Shotgun/Bottom‐Up Proteomics,” Chemical Reviews 113 (2013): 2343–2394. 23438204 10.1021/cr3003533 PMC3751594

[70] V. Lange , P. Picotti , B. Domon , and R. Aebersold , “Selected Reaction Monitoring for Quantitative Proteomics: A Tutorial,” Molecular Systems Biology 4 (2008): 222. 18854821 10.1038/msb.2008.61 PMC2583086

[71] P. Picotti and R. Aebersold , “Selected Reaction Monitoring–Based Proteomics: Workflows, Potential, Pitfalls and Future Directions,” Nature Methods 9 (2012): 555–566. 22669653 10.1038/nmeth.2015

[72] “Method of the Year 2012,” Nature Methods 10 (2013): 1. 23547284 10.1038/nmeth.2329

[73] T. Sequeiros , M. Rigau , C. Chiva , et al., “Targeted Proteomics in Urinary Extracellular Vesicles Identifies Biomarkers for Diagnosis and Prognosis of Prostate Cancer,” Oncotarget 8 (2017): 4960–4976. 27903962 10.18632/oncotarget.13634 PMC5354884

[74] A. C. Peterson , J. D. Russell , D. J. Bailey , M. S. Westphall , and J. J. Coon , “Parallel Reaction Monitoring for High Resolution and High Mass Accuracy Quantitative, Targeted Proteomics,” Molecular & Cellular Proteomics 11 (2012): 1475–1488. 22865924 10.1074/mcp.O112.020131 PMC3494192

[75] N. Rauniyar , “Parallel Reaction Monitoring: A Targeted Experiment Performed Using High Resolution and High Mass Accuracy Mass Spectrometry,” International Journal of Molecular Sciences 16 (2015): 28566–28581. 26633379 10.3390/ijms161226120 PMC4691067

[76] G. E. Ronsein , N. Pamir , P. D. Von Haller , et al., “Parallel Reaction Monitoring (PRM) and Selected Reaction Monitoring (SRM) Exhibit Comparable Linearity, Dynamic Range and Precision for Targeted Quantitative HDL Proteomics,” Journal of Proteomics 113 (2015): 388–399. 25449833 10.1016/j.jprot.2014.10.017 PMC4259393

[77] M. Lin , F. Xu , J. Sun , et al., “Integrative Multi‐Omics Analysis Unravels the Host Response Landscape and Reveals a Serum Protein Panel for Early Prognosis Prediction for ARDS,” Critical Care (London, England) 28 (2024): 213. 38956604 10.1186/s13054-024-05000-3 PMC11218270

[78] A. Martínez‐Val , K. Fort , C. Koenig , et al., “Hybrid‐DIA: Intelligent Data Acquisition Integrates Targeted and Discovery Proteomics to Analyze Phospho‐Signaling in Single Spheroids,” Nature Communications 14 (2023): 3599. 10.1038/s41467-023-39347-y PMC10276052 37328457

[79] L. Breiman , “Statistical Modeling: The Two Cultures (With Comments and a Rejoinder by the Author),” Statistical Science 16 (2001): 199–215.

[80] R. J. Paproski , D. Pink , D. L. Sosnowski , C. Vasquez , and J. D. Lewis , “Building Predictive Disease Models Using Extracellular Vesicle Microscale Flow Cytometry and Machine Learning,” Molecular Oncology 17 (2023): 407–421. 36520580 10.1002/1878-0261.13362 PMC9980304

[81] M. W. Kim , J. Y. Kim , Y. Kim , et al., “Integrating Machine Learning With Microfluidic Technologies for Proteomic Profiling of Extracellular Vesicles in Triple‐Negative Breast Cancer,” Cancer Research 84 (2024): 1843–1843.

[82] M. Bukva , G. Dobra , E. Gyukity‐Sebestyen , et al., “Machine Learning‐Based Analysis of Cancer Cell‐Derived Vesicular Proteins Revealed Significant Tumor‐Specificity and Predictive Potential of Extracellular Vesicles for Cell Invasion and Proliferation—A Meta‐Analysis,” Cell Commun. Signaling 21 (2023): 333. 10.1186/s12964-023-01344-5 PMC10658864 37986165

[83] J. Sun , Z. Li , Y. Chen , Y. Chang , M. Yang , and W. Zhong , “Enhancing Analysis of Extracellular Vesicles by Microfluidics,” Analytical Chemistry 97 (2025): 6922–6937. 40133233 10.1021/acs.analchem.4c07016

[84] K. S. Yang , D. Ciprani , A. O'shea , et al., “Extracellular Vesicle Analysis Allows for Identification of Invasive IPMN,” Gastroenterology 160 (2021): 1345–1358.e11. 33301777 10.1053/j.gastro.2020.11.046 PMC7956058

[85] Y. Zhu , P. D. Piehowski , R. Zhao , et al., “Nanodroplet Processing Platform for Deep and Quantitative Proteome Profiling of 10–100 Mammalian Cells,” Nature Communications 9 (2018): 882. 10.1038/s41467-018-03367-w PMC5830451 29491378

[86] J. A. Bubis , T. N. Arrey , E. Damoc , et al., “Challenging the Astral Mass Analyzer to Quantify up to 5,300 Proteins per Single Cell at Unseen Accuracy to Uncover Cellular Heterogeneity,” Nature Methods 22 (2025): 510–519. 39820751 10.1038/s41592-024-02559-1 PMC11903296

